# A exploratory pilot method comparison study of two viscoelastic coagulation analyzers

**DOI:** 10.3389/fmed.2026.1874412

**Published:** 2026-08-07

**Authors:** Daniel Ricardo Argumedo, Júlio Gallinaro Maranho, Martha R. Ortiz-Posadas

**Affiliations:** 1 Center of Natural and Human Science, Federal University of ABC, Santo André, Brazil; 2 Engineering, Modelling and Applied Social Sciences Center, Federal University of ABC, São Bernardo do Campo, Brazil; 3 Department of Electrical Engineering, Universidad Autónoma Metropolitana-Iztapalapa, Mexico City, Mexico

**Keywords:** viscoelastic hemostatic assay, hemostasis analyzer, thromboelastrometry, method comparison, Bland–Altman analysis, point of care coagulation testing

## Abstract

This preliminary exploratory study evaluated the analytical performance of two different brand of viscoelastic coagulation analyzers in real-time blood samples from ten anonymous patients were analyzed on both devices, and the results were compared using Spearman correlation, Wilcoxon paired testing, Passing–Bablok regression, Bland–Altman agreement analysis, and Lin's concordance correlation coefficient. Systematic biases and variable concordance were observed across several parameters, indicating that the current exploratory dataset does not support direct numerical interchangeability between the two platforms without further testing in larger groups. As new platforms enter the global market, method-comparison studies using agreement-based statistical frameworks should be considered.

## 1 Introduction

Viscoelastic hemostatic assays (VHAs) are point-of-care methods used to assess the whole-blood coagulation process, providing a dynamic picture of clot formation, clot strength, and clot dissolution that conventional coagulation tests cannot provide (1). By testing whole blood rather than plasma, VHAs evaluate the interaction between platelets and the coagulation cascade in real time, making them highly valuable for guiding individualized, goal-directed blood component therapy, particularly in cases of massive hemorrhage (2).

Two broad families of VHA technology are currently in clinical use. Rotational thromboelastometry (ROTEM) systems employ a pin that oscillates within a fixed blood-filled cup; clot formation impedes pin motion, and the signal is captured optically. Thromboelastography (TEG) systems, by contrast, use a rotating cup in which clot formation mechanically couples the cup to a torsion-wire–suspended pin, with the signal transduced electronically. Despite sharing the goal of characterizing whole-blood viscoelasticity, these platforms differ in oscillation mechanics, reagent chemistry, and signal processing, which may produce systematically different numerical outputs for the same clinical sample (3).

VHAs have established clinical applications across a wide range of medical specialties, including cardiac surgery, obstetrics, pediatrics, trauma and emergency medicine, orthopedic surgery, liver transplantation, oncology, sepsis, hemophilia, neurosurgery, urology, and ischemic stroke, among others (4–9). As the number of commercially available VHA platforms grows globally, particularly with the emergence of new devices from manufacturers outside Europe and North America, the analytical equivalence between devices from different technology families can no longer be assumed. Previous comparative studies between established VHA platforms have consistently reported strong correlations but limited interchangeability, with systematic differences attributable to reagent composition, clot-detection mechanisms, and calibration algorithms (10). However, data comparing newer-generation analyzers based on TEG principles against established ROTEM-principle instruments remain scarce.

The objective of this research was to conduct a exploratory pilot method comparison study of two viscoelastic coagulation analyzers operating on different detection principles, a ROTEM-principle device (ROTEM Delta) and a TEG-principle device (Haema TX), both devices use different technologies, including device design, reagent composition, activation methods, platelet inhibition strategies, signal processing, and clot detection thresholds, using multiple complementary statistical approaches to characterize their analytical relationship and assess interchangeability between the two platforms laying the groundwork for subsequent large-scale comparison studies.

## 2 Materials and methods

### 2.1 ROTEM delta

The ROTEM Delta (Werfen, formerly Instrumentation Laboratory; released 2010) is a European, four-channel, manual rotational thromboelastometry system. Its operating principle is based on a pin that oscillates through an arc of 4°45′ every 6 seconds while the blood sample remains in a fixed cup. Clot formation progressively impedes pin oscillation, and this mechanical change is detected by an optical system using a light beam and mirror. The resulting signal is converted into a graphical tracing of clot formation from initiation through maximum firmness and eventual lysis (11). The reagents and measured parameters for this device are shown in (Table 1).

**Table 1:** ROTEM Delta assay and measured parameters.

Assay	Parameters	Activation mechanism
IN-TEM	CT, CFT, MCF, ML, α, LI30	Intrinsic pathway; negatively charged surface (ellagic acid)
EX-TEM	CT, CFT, MCF, ML, α, LI30	Extrinsic pathway; tissue factor activation
FIB-TEM	MCF	Platelet inhibitor (cytochalasin D); isolates fibrinogen contribution

CT: time to initial clot formation.

CFT: time from initial clot to firmness of 20 mm.

MCF: maximum clot firmness; reflects platelet–fibrinogen interaction.

α angle: speed of clot formation.

ML: maximum lysis.

LI30: lysis index at 30 min.

### 2.2 Haema TX

The Haema TX (Medcaptain Medical Technology Co., Ltd., China; released 2021) is a fully automatic, 12-channel thromboelastography system. Its operating principle is mechanical: the blood-filled cup rotates through an arc of approximately 4°45′ every 5–10 seconds (per manufacturer specifications). As clot formation progresses, mechanical coupling between the rotating cup and the torsion-wire–suspended pin increases, transmitting torque to a transducer. The resulting electronic signal is transformed into a thromboelastographic tracing. A disposable inner reaction cup is used for each test to detect blood viscosity changes (12). The reagents and measured parameters for this device are shown in (Table 2).

**Table 2:** Haema TX assay and measured parameters.

Assay	Parameters	Activation mechanism
Kaolin	R, K, α, MA, Ly30	Intrinsic and common pathways; kaolin contact activation
R-Kaolin	R/ACT, K, α, MA, Ly30	Intrinsic and extrinsic pathways; combined activation
FIB	MA	Fibrin-based clot strength; platelet aggregation inhibited

R/ACT: time until initial fibrin formation.

K: time for clot to reach defined firmness.

α angle: speed of clot development.

MA: maximum clot strength; strongly dependent on platelet function.

Ly30: percentage lysis at 30 min.

### 2.3 Experimental procedure

Residual, fully anonymised whole-blood samples were used. These were leftover clinical material remaining after completion of routine care, obtained from a private clinical laboratory in the city of Rio de Janeiro, Brazil. The instruments, reagents, and consumables used in this study were donated by the manufacturers; the donors had no role in study design, sample selection, data analysis, interpretation, or manuscript preparation. Fresh whole-blood samples from ten individuals (five men and five women) were processed in November 2023. Blood samples were collected into 3.2% sodium citrate tubes and analyzed simultaneously on both devices using the same specimen. The interval between blood collection and analysis did not exceed the 4-hour limit recommended by the manufacturers. Reagents for both systems were stored at 2-8 °C according to the manufacturers' instructions.

For ROTEM Delta analysis, citrated blood samples were prewarmed at 37°C in the sample preheating station for 5-10 minutes before testing. Using the device's manual pipette, 300 μl of prewarmed citrated blood was dispensed into the test cup containing 20 μl of STAR-TEM reagent and 20 μl of the corresponding assay reagent. For the INTEM assay, blood was mixed with INTEM reagent; for the EXTEM assay, with EXTEM reagent; and for the FIBTEM assay, with FIBTEM reagent.

For Haema TX analysis, kaolin activator, rapid kaolin activator, fibrinogen activator, trigger solution, and reaction cups were preloaded into their designated compartments within the instrument. Whole blood samples collected in sodium citrate tubes were loaded into the device sample rack. The desired assay (kaolin, rapid kaolin, or fibrinogen) was selected through the instrument touchscreen interface. Haema TX is a fully automated system and does not require manual pipetting during test preparation.

Quality control procedures were performed on both devices prior to sample analysis. ROTROL N and ROTROL P controls were run on the ROTEM Delta system, whereas Control I and Control II were performed on the Haema TX system. All control measurements met the predefined acceptance criteria. In addition, both instruments had current electronic calibration status at the time of testing.

Samples were analyzed simultaneously on both analyzers. The sample size (*n* = 10) was constrained by the high unit cost of viscoelastic testing, the logistical requirements for the parallel operation of two platforms in the same facility and the limited availability of trained personnel. This sample size is not intended to provide statistical power for confirmatory inference; rather, it is consistent with the feasibility scope of the study and with precedent in pilot method-comparison investigations for this technology. Because the samples were residual anonymised material, they were not pre-selected to span any predefined range of coagulation status; the observed range for each parameter is therefore reported together with the summary statistics in the Results, and all agreement estimates should be regarded as valid only across the range of values actually sampled.

### 2.4 Statistical analysis

All statistical analyses were performed using R (version 4.5.2) (13). Agreement analyses were implemented using the SimplyAgree package (14). Descriptive statistics are reported as median with interquartile range (25th–75th percentile) for each device separately; paired within-subject differences (ROTEM Delta minus Haema TX, computed sample-by-sample and then summarized as the median of these ten differences) are reported alongside the two device-level medians and do not necessarily equal the difference between them when distributions are skewed. Differences between the two devices were evaluated using the Wilcoxon signed-rank test, with *p* < 0.05 considered statistically significant.

Monotonic associations between paired measurements were assessed using Spearman's rank correlation coefficient (*r*_*s*_). Agreement between the two systems was evaluated by Bland–Altman analysis (15, 16), in which the bias (mean difference, ROTEM Delta minus Haema TX) and 95% limits of agreement (LoA; bias ± 1.96 SD) were calculated together with their 95% confidence intervals. Because the within-pair differences could not be assumed normally distributed at this sample size, the distribution of differences for each parameter was inspected (Shapiro–Wilk test and visual inspection of the difference plots); where the normality assumption underlying the parametric ±1.96 SD limits was not supported, the corresponding LoA are reported as approximate and are accompanied by the empirical (percentile-based) range of the observed differences. Lin's concordance correlation coefficient (CCC) (17) was computed in its agreement (U-statistic) form to quantify overall agreement, combining both precision and accuracy into a single index; 95% confidence intervals were obtained as implemented in the SimplyAgree package. Values of CCC ≥0.90 were considered excellent, 0.71–0.90 good, 0.51–0.70 moderate, and ≤ 0.50 weak, following a commonly used interpretive convention for concordance.

**Figure 1:**
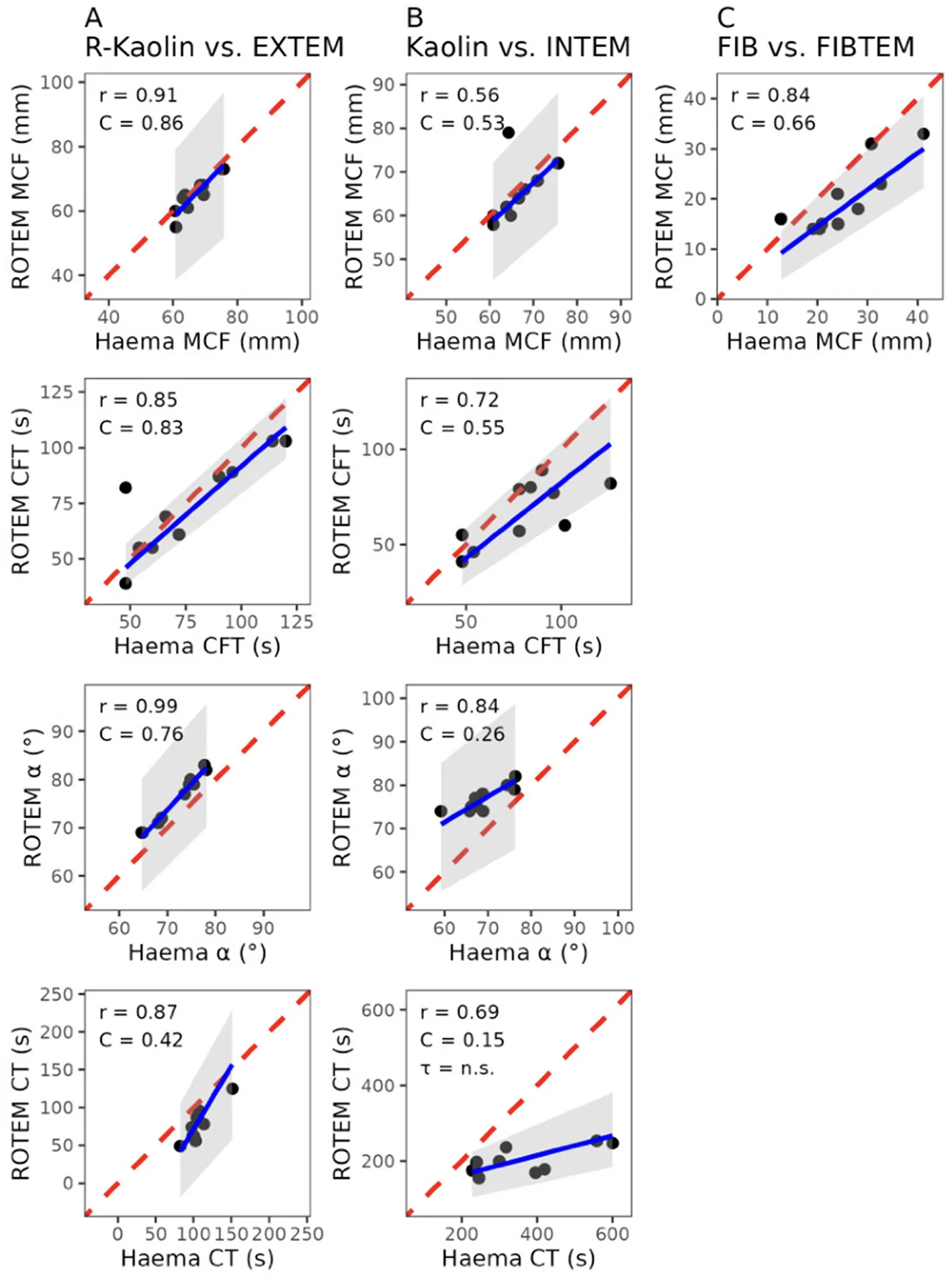
Scatter plots with Passing–Bablok regression lines comparing Haema TX and ROTEM Delta parameters. **(A)** R-Kaolin (Haema TX) vs. EXTEM (ROTEM Delta); **(B)** Kaolin (Haema TX) vs. INTEM (ROTEM Delta); **(C)** FIB (Haema TX) vs. FIBTEM (ROTEM Delta). Within each column, rows show clot formation amplitude (MCF/MA, mm), clot formation time (CFT/K, s), alpha angle (α, °), and clotting time (CT/R, s); the FIB column contains only the MCF panel. Each panel plots Haema TX values (x-axis) against ROTEM Delta values (y-axis), with the Passing-Bablok regression line (blue), its 95% confidence band (gray), and the line of identity (red dashed). Pearson correlation (r) and Lin's concordance (C) coefficients are shown per panel; γ = n.s. indicates a non-significant Kendall's rank correlation.

Passing–Bablok regression was used to assess proportional and systematic errors between methods. The prerequisite of a significant monotonic relationship was verified using Kendall's τ (18, 19); in cases where τ was non-significant, Passing–Bablok regression was not applied. Results are reported as regression intercept and slope with their 95% confidence intervals. The absence of systematic bias was concluded when the 95% CI of the intercept included zero and the 95% CI of the slope included one. Pearson's *r* is additionally reported alongside the Passing–Bablok regression lines in Figure 1 because it is the correlation coefficient conventionally co-displayed with this regression in the method-comparison literature and reflects the linear, rather than purely rank-based, association assumed by the regression model; Spearman's *r*_*s*_ is used in Table 3 as a distribution-free summary statistic that does not require this linearity assumption and is more robust to the small sample size and potential outliers. The two coefficients are not interchangeable and are reported for these distinct purposes rather than as redundant measures of the same quantity.

**Table 3:** Differences and correlations between Haema TX and ROTEM Delta.

Parameter	*n*	Haema	ROTEM	Δ^*^	*p*-val.^†^	*r*_*s*_: 0.83	*p*-val.^‡^: 0.003
R-Kaolin vs. EXTEM							
R/ACT vs. CT (s)	10	103.5 (99.0; 106.9)	76.0 (62.0; 87.8)	−29.9 (−36.3; −19.2)	0.006	0.81	0.004
K vs. CFT (s)	10	69.0 (55.5; 94.5)	75.5 (56.5; 88.5)	−6.0 (−10.5; 0.0)	0.168	
α (°)	10	74.0 (68.3; 75.3)	78.0 (71.2; 79.8)	4.0 (3.4; 4.4)	0.006	0.96	< 0.001
MA vs. MCF (mm)	10	66.5 (63.2; 69.4)	65.0 (61.8; 68.0)	−1.6 (−3.4; 0.6)	0.053	0.89	< 0.001
Kaolin vs. INTEM							
R vs. CT (s)	10	309.0 (241.5; 414.0)	187.5 (174.2; 226.8)	−96.0 (−238.2; 65.5)	0.006	0.56	0.090
K vs. CFT (s)	10	81.0 (60.0; 94.5)	68.5 (55.5; 79.8)	−7.5 (−20.5; 1.8)	0.032	0.73	0.016
α (°)	10	68.2 (66.3; 73.0)	76.0 (74.2; 78.8)	7.9 (5.6; 9.1)	0.006	0.78	0.008
MA vs. MCF (mm)	10	64.5 (61.5; 67.7)	63.0 (60.0; 67.5)	−2.3 (−2.9; 1.0)	0.083	0.74	0.015
FIB vs. FIBTEM							
MA vs. MCF(mm)	10	24.1 (20.5; 30.1)	17.0 (15.0; 22.5)	−6.1 (−8.9; −3.5)	0.019	0.80	0.005

Haema TX and ROTEM Delta columns show each device's own median (25–75th percentile) across the ten samples.

^*^ Δ is the median of the ten paired within-subject differences

(ROTEM Delta − Haema TX for each sample), not the difference between the two device

medians; the two need not coincide for skewed distributions.

^†^ Wilcoxon signed-rank test, uncorrected for multiple comparisons.

^‡^ Spearman correlation, uncorrected for multiple comparisons.

No correction for multiple comparisons was applied to any of the statistical tests reported in this study (Wilcoxon, Spearman, Kendall's τ, or the confidence intervals derived from Bland–Altman, Lin's CCC, and Passing–Bablok analyses). Given the exploratory, hypothesis-generating nature of this pilot comparison and the small sample size (*n* = 10), all reported *p*-values and interval estimates should be interpreted as exploratory and descriptive rather than confirmatory, and are not intended to support formal hypothesis testing or definitive claims of interchangeability for any single parameter in isolation.

## 3 Results

Ten paired fresh whole-blood samples were analyzed simultaneously on both viscoelastic coagulation analyzers, ROTEM Delta and Haema TX, across three assay comparisons: R-Kaolin vs. EXTEM, Kaolin versus INTEM, and FIB versus FIBTEM. Given the small sample size, the full paired raw values for all ten samples and all reported parameters, together with the observed minimum–maximum range for each parameter on each device, are provided in the Supplementary Tables S1–S3, corresponding to the R-Kaolin/EXTEM, Kaolin/INTEM, and FIB/FIBTEM panels, respectively) so that readers can independently verify the analyses, confirm the range of values over which the agreement estimates are valid, and inspect individual data points, including potential outliers, that underlie the summary statistics presented below. The results are presented as follows.

### 3.1 Spearman rank correlation

To characterize the central tendency of each device's measurements and to quantify the monotonic association between paired results, median values with interquartile ranges and Spearman rank correlation coefficients are reported for all nine parameter pairs (Table 3).

R-Kaolin versus EXTEM. Clotting time (R/ACT vs. CT) was significantly shorter on ROTEM Delta than on Haema TX (median paired difference −29.9 s; *p* = 0.006; the corresponding mean difference used in the Bland–Altman analysis was −28.67 s, see Table 4), with a strong Spearman correlation (*r*_*s*_ = 0.81, *p* = 0.004). Clot formation time (K vs. CFT) showed no statistically significant median difference (−6.0 s; *p* = 0.168), though correlation remained strong (*r*_*s*_ = 0.83, *p* = 0.003). The α angle was consistently higher on ROTEM Delta (+4.0°; *p* = 0.006), with the strongest correlation observed across the entire dataset (*r*_*s*_ = 0.96, *p* < 0.001). Maximum clot firmness (MA vs. MCF) showed a borderline-significant negative bias (−1.6 mm; *p* = 0.053) and strong correlation (*r*_*s*_ = 0.89, *p* < 0.001).

**Table 4:** Bland–Altman analysis: bias and 95% limits of agreement, with Lin's CCC (95% CI).

Parameter	*n*	CCC (95% CI)	Term	Estimate (95% CI)
R-Kaolin vs. EXTEM				
R/ACT vs. CT (s)	10	0.42 (0.05; 0.64)	Bias	−28.67 (−36.73; −20.61)
			Lower LoA	−50.74 (−64.69; −36.79)
			Upper LoA	−6.60 (−20.55; 7.35)
K vs. CFT (s)	10	0.83 (0.43; 0.95)	Bias	−2.50 (−12.61; 7.61)
			Lower LoA	−30.21 (−47.72; −12.69)
			Upper LoA	25.21 (7.69; 42.72)
α (°)	10	0.76 (0.53; 0.81)	Bias	4.03 (3.45; 4.61)
			Lower LoA	2.44 (1.43; 3.44)
			Upper LoA	5.62 (4.62; 6.63)
MA vs. MCF (mm)	10	0.86 (0.62; 0.95)	Bias	−1.89 (−3.55; −0.23)
			Lower LoA	−6.43 (−9.30; −3.56)
			Upper LoA	2.65 (−0.22; 5.52)
Kaolin vs. INTEM				
R vs. CT (s)	10	0.15 (−0.01; 0.24)	Bias	−155.70 (−237.88; −73.52)
			Lower LoA	−380.85 (−523.19; −238.52)
			Upper LoA	69.45 (−72.88; 211.79)
K vs. CFT (s)	10	0.55 (0.14; 0.86)	Bias	−13.80 (−26.37; −1.23)
			Lower LoA	−48.23 (−70.00; −26.47)
			Upper LoA	20.63 (−1.13; 42.40)
α (°)	10	0.26 (0.06; 0.38)	Bias	7.78 (5.43; 10.13)
			Lower LoA	1.33 (−2.74; 5.41)
			Upper LoA	14.23 (10.15; 18.30)
MA vs. MCF (mm)	10	0.53 (0.08; 0.90)	Bias	−0.74 (−4.72; 3.24)
			Lower LoA	−11.64 (−18.52; −4.75)
			Upper LoA	10.16 (3.27; 17.04)
FIB vs. FIBTEM				
MA vs. MCF (mm)	10	0.66 (0.22; 0.80)	Bias	−5.40 (−8.57; −2.23)
			Lower LoA	−14.07 (−19.56; −8.59)
			Upper LoA	3.27 (−2.21; 8.76)

Values are Estimate (95% CI). LoA, limits of agreement (bias ± 1.96 SD);

CCC, Lin's concordance correlation coefficient.

Kaolin versus INTEM. Intrinsic-pathway clotting time (R vs. CT) was markedly shorter on ROTEM Delta (median difference −96.0 s; *p* = 0.006), yet Spearman correlation was only moderate and did not reach significance (*r*_*s*_ = 0.56, *p* = 0.090). Clot formation time (K vs. CFT) showed a significant median difference (−7.5 s; *p* = 0.032) and moderate-to-strong correlation (*r*_*s*_ = 0.73, *p* = 0.016). The α angle was again higher on ROTEM Delta (+7.9°; *p* = 0.006; *r*_*s*_ = 0.78, *p* = 0.008). MA vs. MCF did not reach significance (*p* = 0.083), with moderate correlation (*r*_*s*_ = 0.74, *p* = 0.015).

FIB versus FIBTEM. Fibrinogen-related clot firmness (MA vs. MCF) was significantly lower on ROTEM Delta (median difference −6.1 mm; *p* = 0.019), with strong correlation (*r*_*s*_ = 0.80, *p* = 0.005) (Table 3).

### 3.2 Bland–Altman agreement and Lin's concordance correlation coefficient

To evaluate the agreement between the two systems beyond the monotonic associations described above, bias, 95% limits of agreement (LoA), and Lin's concordance correlation coefficient (CCC) were computed for each parameter pair (Table 4).

R-Kaolin versus EXTEM. Clotting time exhibited a systematic negative bias of −28.67 s (95% CI −36.73 to −20.61), with LoA spanning from −50.74 to −6.60 s, and poor concordance (CCC = 0.42, 95% CI 0.05–0.64). Clot formation time showed a non-significant bias of −2.50 s and the highest concordance in this channel (CCC = 0.83, 95% CI 0.43–0.95). The α angle yielded a significant positive bias of 4.03° (95% CI 3.45–4.61) with narrow LoA (2.44 to 5.62°), reflecting a consistent angular offset; concordance was moderate (CCC = 0.76). Maximum clot firmness demonstrated the best overall agreement in the R-Kaolin/EXTEM channel (bias −1.89 mm; CCC = 0.86, 95% CI 0.62–0.95).

Kaolin versus INTEM. The intrinsic-pathway clotting time produced the largest bias of any parameter (−155.70 s; 95% CI −237.88 to −73.52), with LoA extending from −380.85 to +69.45 s and near-absent concordance (CCC = 0.15, 95% CI −0.01–0.24). Clot formation time showed moderate concordance (CCC = 0.55) and a bias of −13.80 s. The α angle yielded poor concordance (CCC = 0.26) with a bias of 7.78°. MA vs. MCF had moderate concordance (CCC = 0.53) and a non-significant bias of −0.74 mm, though the LoA remained wide (−11.64 to +10.16 mm).

FIB versus FIBTEM. A consistent negative bias of −5.40 mm (95% CI −8.57 to −2.23) was observed, with LoA from −14.07 to +3.27 mm and moderate concordance (CCC = 0.66, 95% CI 0.22–0.80) (Table 4).

### 3.3 Passing–Bablok regression

To assess whether proportional or systematic errors existed between the two measurement systems, Passing–Bablok regression lines were overlaid on scatter plots of paired measurements for each parameter and assay combination, alongside the line of identity (Figure 1). Given the small sample size (*n* = 10), the resulting regression coefficients and confidence intervals are reported as exploratory descriptions of the data rather than as definitive estimates of proportional or systematic error, and should be interpreted with appropriate caution; wide confidence intervals are expected and limit the precision with which deviations from the line of identity can be characterized. Columns represent assay comparisons: A R-Kaolin (Haema TX) vs. EXTEM (ROTEM Delta); B Kaolin (Haema TX) vs. INTEM (ROTEM Delta); C FIB (Haema TX) vs. FIBTEM (ROTEM Delta). Rows represent parameters: clot formation amplitude (MCF/MA, mm), clot formation time (CFT/K, s), alpha angle (α, °), and clotting time (CT/R, s). The blue line represents the Passing-Bablok regression line with 95% confidence band (gray shading); the red dashed line represents the line of identity. Pearson correlation coefficient (r) and Lin's concordance correlation coefficient (C) are shown for each pair, consistent with the convention of co-reporting Pearson's *r* alongside Passing–Bablok regression in the method-comparison literature (see Methods); this differs from the Spearman coefficient (*r*_*s*_) reported in Table 3. τ = n.s. indicates a non-significant Kendall's rank correlation.

R-Kaolin versus EXTEM. The regression lines approached the identity line most closely for MA vs. MCF (*r* = 0.91, *C* = 0.86) and K vs. CFT (*r* = 0.85, *C* = 0.83). The α angle showed near-perfect Pearson correlation (*r* = 0.99) but only moderate concordance (*C* = 0.76), consistent with the systematic offset identified in the agreement analysis. Clotting time showed strong linear correlation (*r* = 0.87) but poor concordance (*C* = 0.42), with the regression line deviating visibly from the identity line; given *n* = 10, this pattern is best read as suggestive rather than conclusive evidence of proportional or constant bias.

Kaolin versus INTEM. The agreement was uniformly lower across all parameters. For intrinsic-pathway clotting time (R vs. CT), Kendall's τ was non-significant, precluding valid Passing–Bablok regression for this parameter; the corresponding panel is annotated accordingly (τ= n.s.). Pearson correlation for this parameter was the lowest across the entire dataset (*r* = 0.69, *C* = 0.15).

FIB versus FIBTEM. The regression demonstrated strong linear correlation (*r* = 0.84) alongside moderate concordance (*C* = 0.66); as elsewhere, this single-channel estimate from ten paired samples should be treated as a preliminary observation pending confirmation in a larger dataset (Figure 1).

### 3.4 Bland–Altman plots

**Figure 2:**
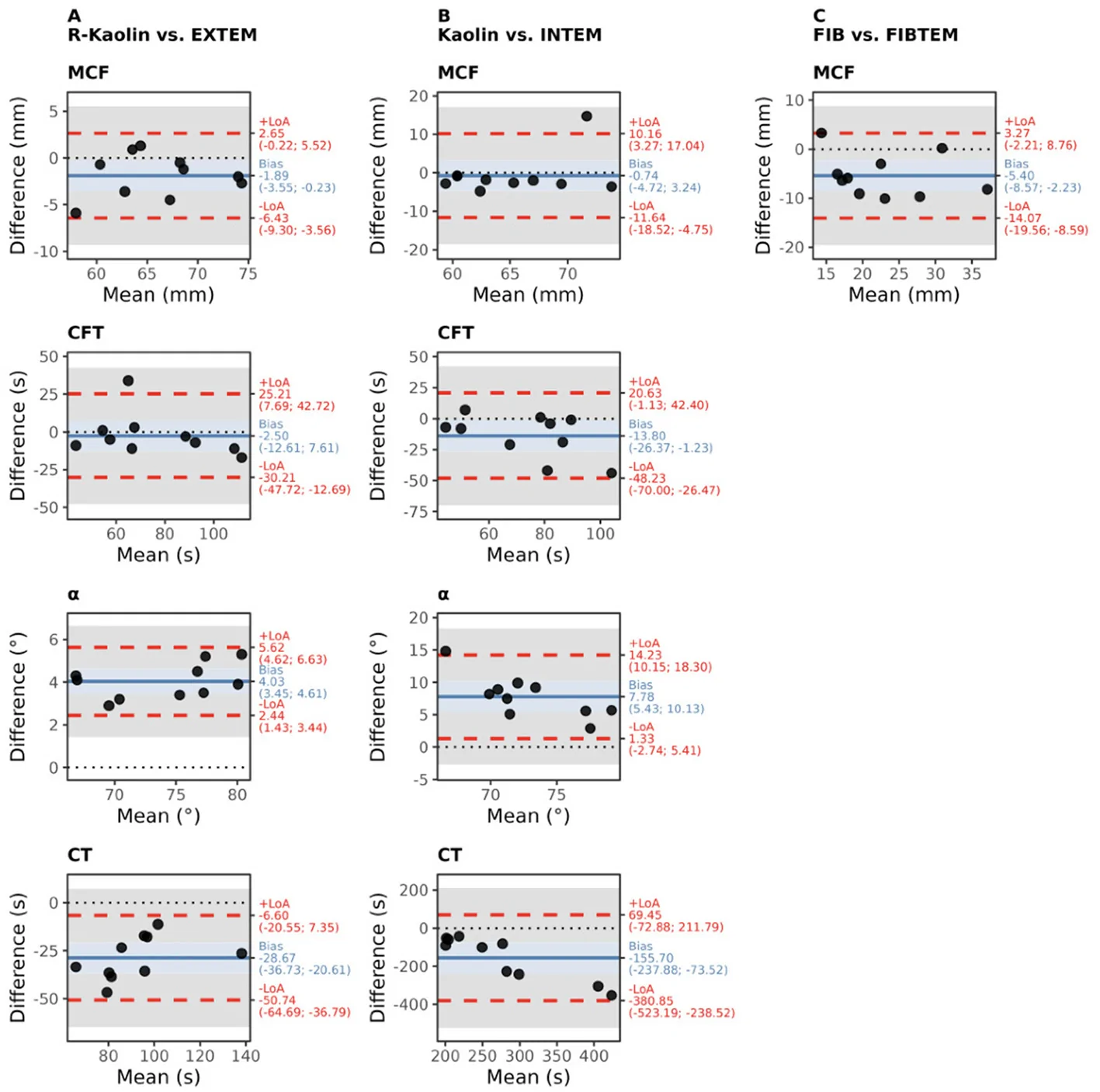
Bland-Altman plots comparing Haema TX and ROTEM Delta parameters. **(A)** R-Kaolin (Haema TX) vs. EXTEM (ROTEM Delta); **(B)** Kaolin (Haema TX) vs. INTEM (ROTEM Delta); **(C)** FIB (Haema TX) vs. FIBTEM (ROTEM Delta). Within each column, rows show clot formation amplitude (MCF, mm), clot formation time (CFT, s), alpha angle (α, °), and clotting time (CT, s); the FIB column contains only the MCF panel. Each panel plots the mean of the two devices (x-axis) against their paired difference, ROTEM Delta minus Haema TX (y-axis), with the mean bias (solid blue) and its 95% confidence interval, the 95% limits of agreement (red dashed) with their confidence intervals, and the zero-difference line (dotted black).

To visualize the distribution of measurement differences relative to mean values and to assess whether bias was constant across the measurement range, Bland–Altman plots were constructed for each parameter pair across all three assay channels (Figure 2). Columns represent assay comparisons: A R-Kaolin (Haema TX) vs. EXTEM (ROTEM Delta); B Kaolin (Haema TX) vs. INTEM (ROTEM Delta); C FIB (Haema TX) vs. FIBTEM (ROTEM Delta). Rows represent parameters: clot formation amplitude (MCF, mm), clot formation time (CFT, s), alpha angle (α, °), and clotting time (CT, s). The x-axis shows the mean of the two methods; the y-axis shows the difference (ROTEM Delta - Haema TX). The solid blue line represents the mean bias with its 95% confidence interval (blue shading); red dashed lines represent the 95% limits of agreement (LoA, bias ± 1.96 SD) with their 95% confidence intervals (gray shading); the dotted black line represents zero difference. Numerical values on the right axis indicate the bias and LoA estimates with their respective 95% confidence intervals. LoA, limits of agreement.

R-Kaolin versus EXTEM. The mean bias line for clotting time lay entirely below zero, with both LoA negative, confirming a consistent direction of disagreement independent of the measurement magnitude. The α angle plots showed a narrow band of differences clustered above zero, with no evident proportional bias across the measurement range. The MA vs. MCF plots displayed differences distributed symmetrically around a small negative bias, with all ten data points within the LoA.

Kaolin versus INTEM. The clotting time plot demonstrated the widest spread of any parameter, with individual differences spanning several hundred seconds and the upper LoA exceeding zero. Several data points fell near or beyond the LoA boundaries, visually corroborating the large uncertainty reported in Table 4. The MA vs. MCF plot, by contrast, showed a tighter distribution around a near-zero bias, though the LoA remained broad (±~11 mm).

FIB versus FIBTEM. The difference plot showed all ten paired observations below zero, consistent with the systematic negative bias of −5.40 mm, with limited scatter around the mean bias line (Figure 2).

## 4 Discussion

This exploratory work evaluated the analytical relationship between two viscoelastic coagulation analyzers operating on different detection principles, the ROTEM Delta (rotational thromboelastometry) and the Haema TX (thromboelastography), both devices have different technology. Including device design, reagent composition, activation methods, platelet inhibition strategies, signal processing, and clot detection thresholds. This study compares different platform-assay combinations, to determine if an interchangeability relationship exists by comparing parameters obtained from equivalent assays across the intrinsic, extrinsic, and fibrinogen-specific pathways.

Before interpreting individual parameters, it is important to emphasize that with only ten paired samples and nine parameter comparisons evaluated without correction for multiple testing, the statistical analyses presented here cannot support firm conclusions about any single parameter in isolation. The combination of Wilcoxon testing, Spearman correlation, Bland–Altman analysis, Lin's CCC, and Passing–Bablok regression is appropriate in principle for characterizing a method-comparison relationship, but applying five complementary methods to a small dataset can create an impression of analytical robustness that the sample size does not support. Accordingly, the discussion below emphasizes the width of the confidence intervals and limits of agreement, and the overall pattern across parameters and assays, rather than the statistical significance of any individual *p*-value, which should be read as exploratory and hypothesis-generating only.

In the R-Kaolin versus EXTEM comparison, the α angle demonstrated very high correlation (*r*_*s*_ = 0.96), and clot firmness (MA vs. MCF) also showed strong correlation (*r*_*s*_ = 0.89). These findings indicate that both analyzers capture similar clot formation kinetics and final clot strength. However, the concordance analysis demonstrates that strong correlation does not imply interchangeability between the two platforms without further testing in larger groups. The CCC for clotting time was low (CCC = 0.42), with a 95% confidence interval (0.05–0.64) wide enough to be compatible with anywhere from poor to moderate agreement, reflecting systematic measurement differences between devices as well as the imprecision inherent to estimating CCC from ten pairs. Such discrepancies may arise from differences in measurement mechanics, technology differences in systems and reagent composition.

The two analyzers employ fundamentally different detection principles. RO-TEM Delta relies on optical detection of pin movement in a fixed cup, whereas Haema TX uses mechanical torsion-wire transduction with a rotating cup configuration. Variations in oscillation frequency, torque transmission, and clot-detection thresholds may influence the sensitivity of each analyzer to early fibrin polymerization and platelet–fibrin interactions. These mechanical differences may contribute to the systematic biases observed in clotting time measurements.

The largest disagreement was observed in the intrinsic pathway comparison (Kaolin vs. INTEM), particularly for clotting time. ROTEM Delta CT values were markedly shorter than the corresponding Haema TX R values, with a large bias of −155.70 s and very wide limits of agreement. The CCC for this parameter was extremely low (CCC = 0.15), indicating near-complete lack of agreement despite moderate correlation. Kendall's τ was non-significant for this parameter, preventing valid Passing–Bablok regression. These findings indicate that intrinsic pathway clot initiation is captured differently by the two analyzers and that numerical results should not be considered equivalent without further testing in larger groups. In contrast, clot firmness parameters demonstrated consistently better agreement across both platforms. MA versus MCF comparisons showed strong correlations and moderate-to-high concordance, particularly in the EXTEM channel (CCC = 0.86). This pattern is consistent with previous comparative studies of viscoelastic platforms, which have reported that devices tend to converge in the measurement of final clot mechanical properties, as these parameters depend primarily on platelet–fibrin interactions rather than on early coagulation cascade dynamics (10).

The fibrinogen-specific assays (FIB vs. FIBTEM) showed strong correlation (*r*_*s*_ = 0.80) but a systematic negative bias, indicating that ROTEM Delta consistently reports lower fibrin-based clot firmness than Haema TX. This difference may reflect variations in platelet-inhibition reagent composition or differences in fibrin polymerization sensitivity between the two platforms.

From a clinical perspective, this exploratory study provides an overview of the correlation and interchangeability between the two viscoelastic coagulation analyzers. The presence of systematic bias and wide limits of agreement indicates that measurements from one device cannot be directly substituted for those from the other without further testing in larger groups.

Clinical algorithms based on viscoelastic testing rely on specific threshold values for parameters such as CT, CFT, and MCF. Applying thresholds derived from one analyzer to another could potentially lead to different clinical classifications if thresholds from one platform are used on another without validation or misclassification of coagulation status. Our results are broadly consistent with the direction of findings from larger comparative studies between viscoelastic platforms, which have similarly identified reagent composition, clot-detection mechanisms, and calibration algorithms as key sources of inter-device variability. Ziegler et al. compared the TEG 6s and ROTEM Sigma analyzers in a prospective cohort of trauma patients and likewise reported strong correlations alongside clinically relevant systematic differences between platforms (10). Infanger et al. compared ClotPro and ROTEM Delta in critically ill patients with COVID-19 and reported that, despite good overall agreement for several parameters, the two devices were not interchangeable, with notable platform-specific differences in fibrinolysis detection (20). Grüneberg et al. compared ClotPro and ROTEM Delta head-to-head across extrinsic, intrinsic, and fibrin-polymerization assays and reported fair-to-moderate correlation for clotting times, apparent interchangeability for clot firmness in extrinsically activated tests, but systematic and clinically relevant differences in fibrin-polymerization assays, with ClotPro consistently yielding higher values than ROTEM Delta (21).

These larger studies, enrolling far more paired samples than the present pilot, converge on the same qualitative conclusion reached here, namely that correlation between viscoelastic platforms does not establish interchangeability, and that clotting-time and fibrinogen-specific parameters are particularly prone to systematic, device-specific bias. Because the present study includes only ten paired samples, however, it should be regarded as contributing weak, hypothesis-generating evidence toward this broader pattern rather than an independent confirmation of it; the device-specific magnitudes reported here are illustrative of the pilot dataset and are not precise enough to be used as correction factors.

The study exploratory employs a comprehensive multi-method statistical framework, including Spearman correlation, Wilcoxon paired testing, Passing–Bablok regression, Bland–Altman agreement analysis, and Lin's CCC, that provides a pilot study of characterization of the analytical relationship between the two analyzers.

The results demonstrate that while Haema TX and ROTEM Delta capture comparable aspects of clot formation dynamics, statistically, no interchangeability relationship was found with the analyzed data. It is recommended that the results of each device should therefore be evaluated using its own validated reference ranges, and future work should focus on establishing device-specific clinical decision thresholds in larger prospective cohorts.

### 4.1 Study limitations

This study should be interpreted considering several limitations. First, the investigation was designed as an exploratory pilot method-comparison study and included a limited number of paired samples, which restricts the statistical precision of agreement estimates. Consequently, the findings should be considered preliminary and require confirmation in larger cohorts.

Second, detailed clinical and laboratory information for the analyzed samples, including coagulation status, hematological parameters, medication use, and other patient characteristics, was not available because the samples were fully anonymized. Similarly, some pre-analytical variables known to influence viscoelastic testing, such as blood collection conditions, transport, and processing times, could not be systematically evaluated.

Third, the assay channels compared between platforms were clinically analogous but not analytically identical, differing in reagent composition, activation mechanisms, clot-detection thresholds, and signal-processing algorithms. Therefore, the observed differences cannot be attributed exclusively to instrument hardware and should instead be interpreted as reflecting the combined effect of platform- and assay-specific characteristics.

Fourth, the study focused exclusively on analytical agreement and did not assess clinical outcomes, transfusion practices, or patient classification based on established therapeutic algorithms.

Fifth, the statistical analyses themselves carry limitations stemming directly from the small sample size. With only ten paired samples, estimates of bias, limits of agreement, and Lin's CCC have wide confidence intervals, and the Passing–Bablok regression coefficients in particular should be interpreted as exploratory descriptions of the data rather than precise estimates of proportional or systematic error. No correction for multiple comparisons was applied across the nine parameter pairs and five analytical approaches used, so the reported *p*-values should not be used to draw firm conclusions about any single parameter and are presented for hypothesis-generating purposes only. To allow independent verification of these analyses and inspection of individual data points, the full paired raw values for all ten samples are provided in (Supplementary Tables S1–S3).

Finally, no predefined criteria for acceptable agreement were established, and the single-center nature of the study may limit the generalizability of the results. Future multicenter investigations involving larger and clinically diverse populations will be necessary to validate these observations and determine their clinical relevance.

## 5 Conclusion

This pilot study demonstrated a preliminary exploratory observation between the ROTEM Delta and the Haema TX, although measuring comparable aspects of whole-blood coagulation. The current dataset does not support direct numerical interchangeability between the two platforms without furthertesting in larger groups.

Systematic biases and variable concordance were identified across all assay channels, indicating that numerical results from one platform cannot be directly substituted for those from the other for this data that require confirmation in larger cohorts.

These findings underscore a broader principle in the evaluation of viscoelastic coagulation technology: correlation is not equivalence. As new platforms enter the global market, method-comparison studies using agreement-based statistical frameworks should be considered a prerequisite for clinical adoption, rather than an afterthought. Subsequent prospective investigations in larger, clinically diverse cohorts will be necessary to establish whether device-specific conversion models are feasible and to support evidence-based implementation of emerging viscoelastic analyzers.

Future research should prioritize multicenter comparative studies to assess the repeatability and reproducibility of viscoelastic testing across different institutions using the same devices. Such investigations are essential to quantify inter-center variability and establish the robustness of these technologies in diverse clinical settings. Moreover, as the number of viscoelastic testing platforms continues to expand, there is an increasing need for international standardization. The development of a universal reference metric, analogous to the International Normalized Ratio (INR) for prothrombin time, could facilitate harmonized interpretation of abnormal viscoelastic results, improve comparability between devices, and ultimately support more consistent and reliable clinical decision-making worldwide.

## Data Availability

The original contributions presented in the study are included in the article/Supplementary material, further inquiries can be directed to the corresponding author.
