# Supplementary material for: A exploratory pilot method comparison study of two viscoelastic coagulation analyzers
Source: Front Med (Lausanne). 2026 Aug 7;13:1874412. doi: 10.3389/fmed.2026.1874412 (PMC13493386; doi:10.3389/fmed.2026.1874412)
Supplement: Supplementary file 1 [file Data_Sheet_1.pdf]

Supplementary Material

A Exploratory Pilot Method Comparison Study of Two Viscoelastic Coagulation Analyzers

Daniel Ricardo Argumedo Júlio Gallinaro Maranho Martha R. Ortiz-Posadas

This supplementary material reports the raw, unprocessed measurements obtained from the two viscoelastic coagulation analyzers (Haema TX and ROTEM Delta) for each of the ten anonymous patients, across the three assay panels evaluated: R-Kaolin INTEM, R-Kaolin EXTEM, and FIB FIBTEM. These values were used as input for the statistical analyses (Spearman correlation, Wilcoxon paired testing, Passing–Bablok regression, Bland–Altman agreement analysis, and Lin's concordance correlation coefficient) presented in the main text.

Table 1: R-Kaolin EXTEM — Raw Values

ID	Haema RACT (s)	ROTEM CT (s)	Haema K (min)	ROTEM CFT (s)	Haema α (°)	ROTEM α (°)	Haema MA (mm)	ROTEM MCF (mm)
1	100.5	62	2.0	103	64.7	69	60.7	60
2	102.7	56	1.0	55	74.5	79	69.2	68
3	82.4	49	1.2	61	74.8	80	68.5	68
4	105.8	88	1.6	89	68.8	72	63.7	65
5	113.6	78	1.1	69	73.6	77	69.5	65
6	97.4	74	0.9	55	75.5	79	75.7	73
7	98.5	62	1.9	103	64.9	69	64.6	61
8	107.2	96	0.8	82	77.7	83	60.9	55
9	151.4	125	0.8	39	78.1	82	75.0	73
10	104.2	87	1.5	87	68.1	71	63.1	64

Table 2: Kaolin INTEM — Raw Values

ID	Haema R (min)	ROTEM CT (s)	Haema K (min)	ROTEM CFT (s)	Haema α (°)	ROTEM α (°)	Haema MA (mm)	ROTEM MCF (mm)
1	5.0	199	1.7	60	65.8	74	60.8	60
2	3.8	175	0.9	46	74.4	80	68.0	66
3	3.9	174	0.8	55	76.1	79	70.9	68
4	5.3	236	1.3	79	68.9	74	64.3	79
5	6.6	169	1.3	57	68.8	78	66.6	64
6	9.3	253	1.6	77	66.1	75	64.8	60
7	4.1	155	0.8	41	76.3	82	75.6	72
8	10.0	247	2.1	82	59.2	74	60.8	60
9	4.0	197	1.4	80	67.5	75	63.8	62
10	7.0	178	1.5	89	67.1	77	60.8	58

Table 3: FIB FIBTEM — Raw Values

ID	Haema MA (mm)	ROTEM MCF (mm)
1	19.1	14
2	24.1	15
3	24.0	21
4	20.4	14
5	28.1	18
6	32.7	23
7	12.7	16
8	30.8	31
9	41.2	33
10	20.9	15
